# Plaque-associated human microglia accumulate lipid droplets in a chimeric model of Alzheimer’s disease

**DOI:** 10.1186/s13024-021-00473-0

**Published:** 2021-07-23

**Authors:** Christel Claes, Emma Pascal Danhash, Jonathan Hasselmann, Jean Paul Chadarevian, Sepideh Kiani Shabestari, Whitney E. England, Tau En Lim, Jorge Luis Silva Hidalgo, Robert C. Spitale, Hayk Davtyan, Mathew Blurton-Jones

**Affiliations:** 1 Department of Neurobiology and Behavior, University of California Irvine, Irvine, CA 92696 USA; 2 Sue and Bill Gross Stem Cell Research Center, University of California Irvine, Irvine, CA 92696 USA; 3 Department of Neurobiology and Behavior, University of California Irvine, Irvine, CA 92696 USA; 4 Department of Pharmaceutical Sciences, University of California, Irvine, CA 92697 USA; 5 Center for the Neurobiology of Learning and Memory, University of California, Irvine, CA 92697 USA

**Keywords:** TREM2, Human microglia, Chimeric Alzheimer mice, Lipid droplets

## Abstract

**Background:**

Disease-associated microglia (DAMs), that surround beta-amyloid plaques, represent a transcriptionally-distinct microglial profile in Alzheimer’s disease (AD). Activation of DAMs is dependent on triggering receptor expressed on myeloid cells 2 (TREM2) in mouse models and the AD TREM2-R47H risk variant reduces microglial activation and plaque association in human carriers. Interestingly, TREM2 has also been identified as a microglial lipid-sensor, and recent data indicates lipid droplet accumulation in aged microglia, that is in turn associated with a dysfunctional proinflammatory phenotype. However, whether lipid droplets (LDs) are present in human microglia in AD and how the R47H mutation affects this remains unknown.

**Methods:**

To determine the impact of the TREM2 R47H mutation on human microglial function *in vivo*, we transplanted wild-type and isogenic TREM2-R47H iPSC-derived microglial progenitors into our recently developed chimeric Alzheimer mouse model. At 7 months of age scRNA-seq and histological analyses were performed.

**Results:**

Here we report that the transcriptome of human wild-type TREM2 and isogenic TREM2-R47H DAM xenografted microglia (xMGs), isolated from chimeric AD mice, closely resembles that of human atherosclerotic foam cells. In addition, much like foam cells, plaque-bound xMGs are highly enriched in lipid droplets. Somewhat surprisingly and in contrast to a recent *in vitro* study, TREM2-R47H mutant xMGs exhibit an overall reduction in the accumulation of lipid droplets *in vivo*. Notably, TREM2-R47H xMGs also show overall reduced reactivity to plaques, including diminished plaque-proximity, reduced CD9 expression, and lower secretion of plaque-associated APOE.

**Conclusions:**

Altogether, these results indicate lipid droplet accumulation occurs in human DAM xMGs in AD, but is reduced in TREM2-R47H DAM xMGs, as it occurs secondary to TREM2-mediated changes in plaque proximity and reactivity.

**Supplementary Information:**

The online version contains supplementary material available at 10.1186/s13024-021-00473-0.

## Findings

Genetic studies strongly implicate microglia in the development and progression of Alzheimer’s disease (AD) [1–5]. In particular, the R47H coding mutation in TREM2 (triggering receptor expressed on myeloid cells 2) is one of the strongest microglial-expressed risk factors, causing a three-fold increase in the development of late-onset AD [6–12]. Moreover, homozygous R47H mutations were shown to further lower the age of disease onset [13]. Recently, single-cell RNA sequencing (scRNA-seq) of an AD mouse model identified disease-associated microglia (DAMs), a population of plaque-adjacent microglia that exhibit a distinct TREM2-dependent transcriptional profile [14, 15]. Many other studies have shown a similarly reduced range of microglial responses to neurodegeneration with TREM2 deletion including alterations in proliferation, phagocytosis, cytokine production, plaque association, and survival [16–23]. Overall, these data suggest that TREM2 protects from AD at least in part by enabling microglia to surround and compact beta-amyloid plaques, thereby limiting neuritic dystrophy and other secondary damage [20]. Subsequent *in vivo* studies of a CRISPR-induced murine TREM2 R47H variant, designed to mimic the most common AD TREM2 mutation within the extracellular ligand-binding domain [24], suggested that this mutation may increase AD risk by conferring a partial loss of TREM2 function [25]. However, further analysis revealed that introduction of the R47H variant impaired splicing and reduced Trem2 mRNA and protein in mice, but not in human microglia, suggesting that such mouse models phenocopy heterozygous TREM2 deletions [26]. Nevertheless, analysis of postmortem brain tissue from R47H-carriers confirms that mutant microglia exhibit a markedly reduced ability to envelop amyloid deposits [18] and recent single-nucleus transcriptomics further reveals that this reactive microglial phenotype is less evident in both TREM2-R47H carriers and mutant mice [27].

TREM2 has also been strongly implicated in brain lipid biology. For example, TREM2 binds to and senses lipids and the TREM2-R47H mutation was shown to reduce lipid-binding *in vitro* [20, 28]. Moreover, many other AD risk factors are implicated in microglial lipid metabolism including APOE4, ABCA7, CLU and SLC24A4 [2]. Interestingly, Alois Alzheimer was himself the first to describe “glial cells showing adipose saccules” within the brains of dementia patients in 1907 [29]. Remarkably, very few studies since then have examined the potential role of microglial lipid accumulation in AD or determined whether the R47H TREM2 mutation impacts microglial lipid homeostasis *in vivo* [30–33]. In this respect, one recent study reported the presence of lipid droplet-accumulating microglia (LDAMs) within aged murine and human brains, leading to changes in phagocytic and proinflammatory response [34]. Interestingly, a very similar process has been described within the periphery where excessive storage in lipid droplets (LDs) results in the formation of “foamy macrophages” [35] which is central to the pathogenesis of prevalent metabolic diseases, including obesity, diabetes, and atherosclerosis, all of which are risk factors for AD [36]. Yet, LDAMs exhibit a phenotype which differs markedly from DAMs; while DAMs are actively phagocytic and show a TREM2-dependent transcriptome, LDAMs are severely impaired in phagocytosis and their transcriptomic signature is TREM2-independent [34].

Interestingly, human microglia that overexpress the R47H mutation *in vitro*, appear to be metabolically challenged, exhibiting excessive lipid accumulation after chronic myelin treatment compared to wild-type TREM2 expressing counterparts [37]. Therefore, to study whether LD accumulation occurs in human microglia *in vivo* and determine the impact of the R47H-mutation on this process, we combined isogenic iPSC-derived human microglia with a recently established chimeric mouse model of AD [38]. By transplanting wild-type and R47H human microglial progenitors into 5x-hCSF1 mice [38], we examined the impact of this mutation on lipid droplet accumulation, the microglial response to beta-amyloid plaques, and human microglia gene expression of isolated xenografted iPSC-derived microglia (xMGs).

### Single cell transcriptomic analysis reveals DAM xMGs resemble atherosclerotic foam cells and signature gene expression reveals partial TREM2 dependence

**Fig. 1:**
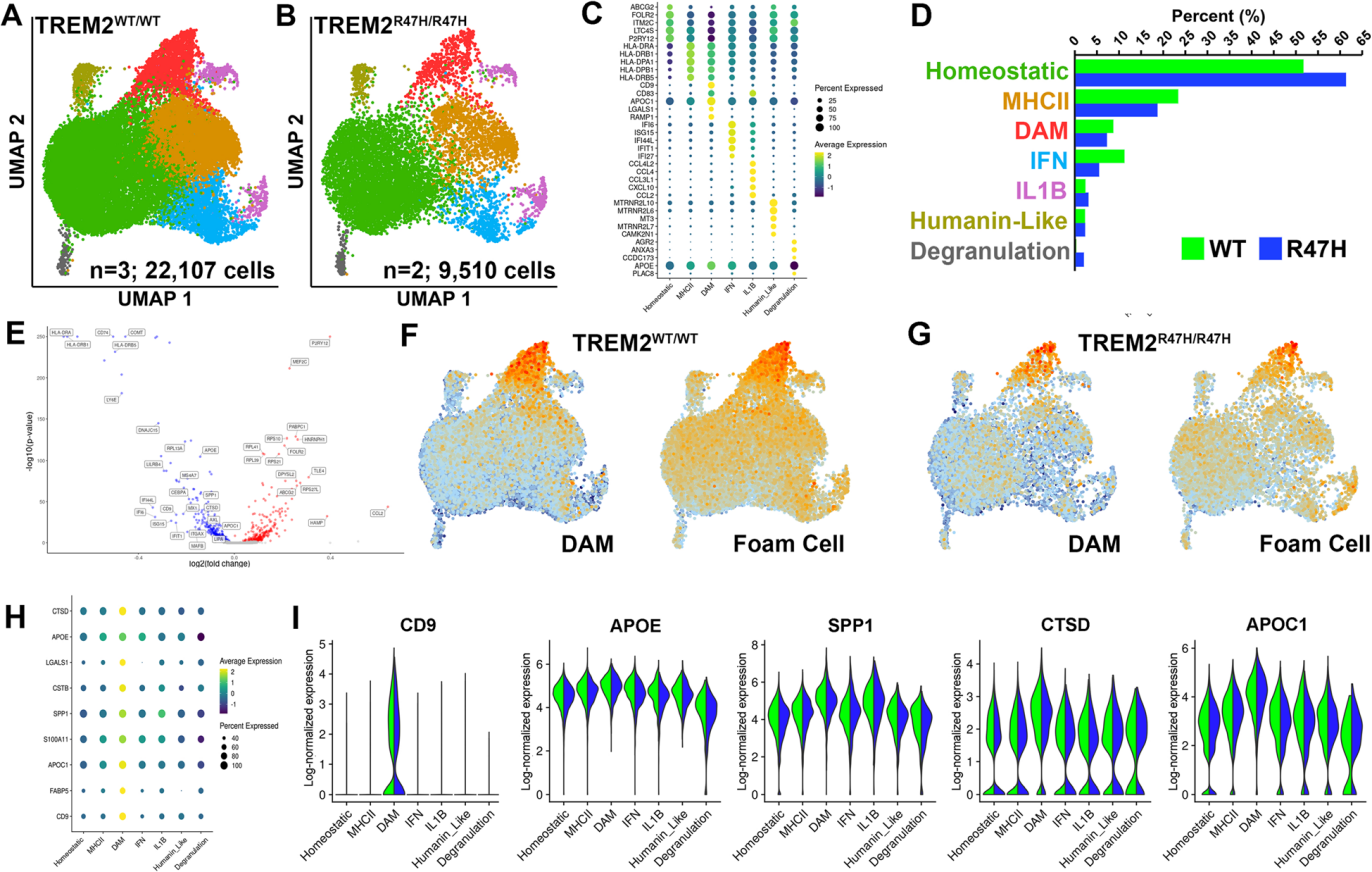
Disease associated microglia resemble atherosclerotic foam cells and signature gene expression reveals partial TREM2 dependence (**A**) UMAP plot displaying clustering of TREM2^WT/WT^xMGs isolated from 7-month-old 5X-hCSF1 mice (*n*=3 mice; 22,107 cells; WT xMGs per mouse (5987, 7876, 8244) and R47H xMGs per mouse (3939, 5571); **B** UMAP plot displaying clustering of TREM2^R47H/R47H^xMGs isolated from 7-month-old 5X-hCSF1 mice (*n*=2 mice; 9,510 cells); **C** A dot plot reveals top marker genes that are significantly up- or downregulated for each of the seven clusters (FDR-adjusted *p*-value < 0.01); (**D**) Barplot displaying the average percentage of cells making up the 7 unique xMG clusters; **E** Volcano plot displaying genes significantly up- and downregulated when comparing all TREM2^R47H/R47H^ cells vs. all TREM2^WT/WT^ cells; **F-G** UMAP plots displaying the module scores for 7 key DAM signature genes and 29 Foam cell signature genes identified in (Fernandez*et al.*, [41]) (Additional File 1) in TREM2^WT/WT^ (**F**) and TREM2^R47H/R47H^(**G**); **H** A dot plot reveals foam cell genes are particularly enriched in the DAM cell cluster (FDR-adjusted *p*-value < 0.01). Size of the circles indicate the percentage of cells expressing that gene and color indicates average expression levels; **I** Violin plots demonstrate foam cell signature genes are particularly enriched in the DAM cell cluster vs. other clusters, and are significantly (adjusted *p*-value < 0.01; LFC≥0.01) albeit subtly downregulated in R47H cells when comparing all TREM2^R47H/R47H^ (blue) vs. all TREM2^WT/WT^ cells (green) via pseudobulk analysis (Additional File 1)

Seven months after transplantation, GFP-expressing TREM2 and isogenic TREM2-R47H xenografted microglia (xMGs) were isolated from 5x-hCSF1 mouse brains and examined via scRNA-sEq. Analysis revealed 7 discrete clusters of xMGs, each representing a unique microglial state for both TREM2 and TREM2-R47H xMGs (Fig. 1A-C). Results show the TREM2-R47H variant induced a slightly enhanced homeostatic (resting) signature with a corresponding decrease in more reactive microglial populations including DAMs, MHCII-enriched, and interferon responsive (IFN) clusters (Fig. 1D), although these changes did not reveal significance by differential proportion analysis (DPA [39], not shown). However, subsequent pseudobulk analysis did reveal significant up- and downregulation of a number of genes, including downregulation of genes involved in lipid metabolism e.g. SPP1, APOE and CTSD in TREM2-R47H xMGs (Fig. 1E, Additional File 1). As TREM2 is a known lipid-sensing receptor and a recent study demonstrated the presence of lipid droplet-accumulating microglia in aged mice, we next sought to determine whether the transcriptome of xMGs might resemble atherosclerotic foam cells, a lipid-accumulating peripheral macrophage population [20, 34, 40, 41]. Examining the expression levels of 29 genes that were recently identified as being enriched in human foam cells in atherosclerosis [41] (Additional File 1) revealed a pronounced enrichment of foam cell genes within the DAM cluster (Fig. 1F-H) and reveal partial TREM2 dependence with CD9, APOC1, SPP1, CTSD and APOE being most highly expressed within the DAM cluster (Fig. 1H, I) and overall significantly, albeit subtly, downregulated in R47H cells when comparing all TREM2-R47H cells vs. TREM2-WT cells (Fig. 1E, I). This suggests a conserved phenotype between human microglia responding to beta-amyloid plaques within the brain and human foam cells in atherosclerosis within the periphery.

### Plaque-associated human microglia accumulate Lipid Droplets in chimeric mice and AD brains, and Lipid Droplet area is reduced in TREM2-R47H transplanted mice

**Fig. 2:**
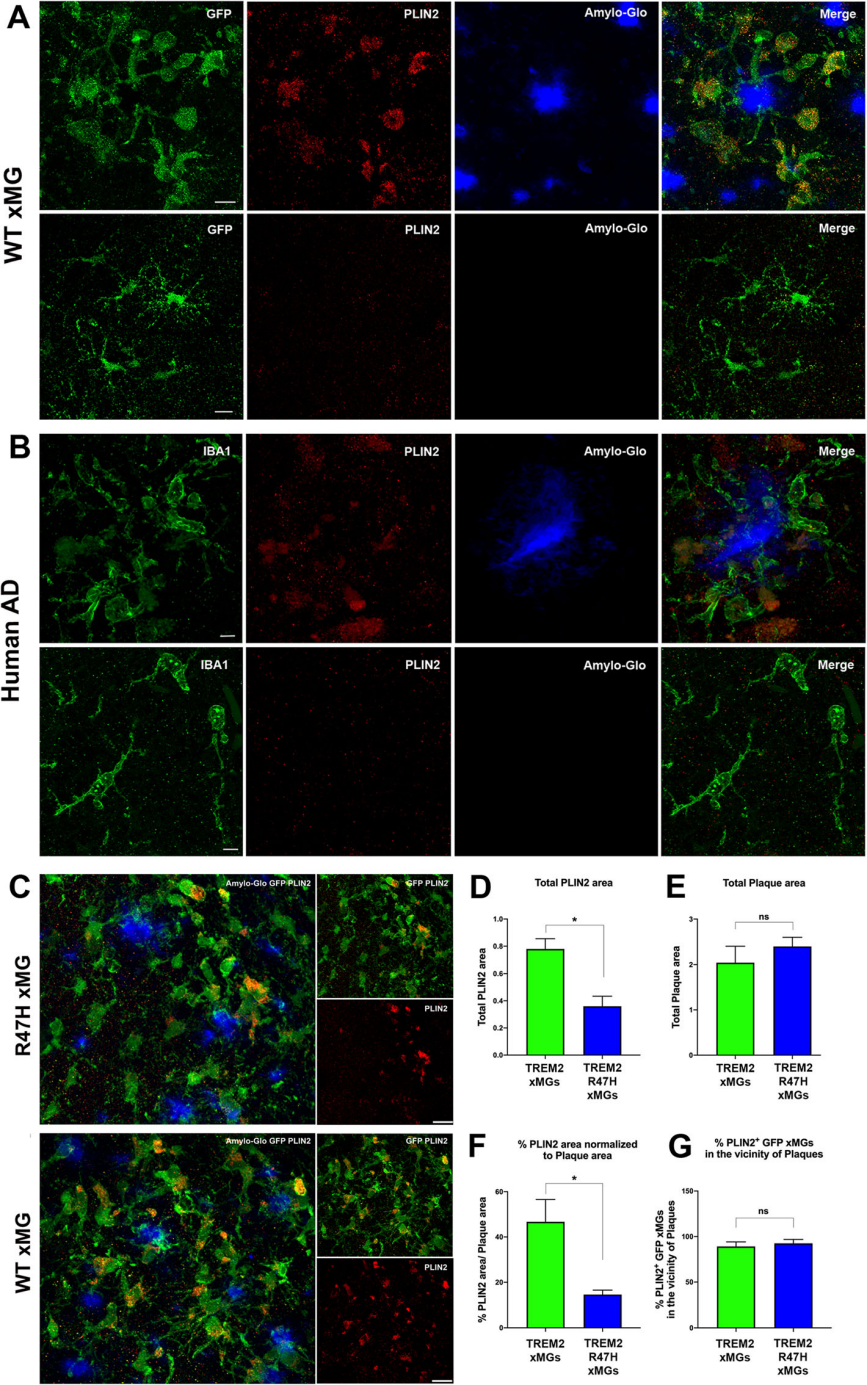
Plaque-associated human microglia accumulate Lipid Droplets, and Lipid Droplet area is reduced in TREM2-R47H transplanted mice. **A** Lipid droplets (red, PLIN2) accumulate in human microglia (green, GFP) adjacent to plaques in chimeric mice (blue, Amylo-Glo), but not in microglia distant from plaques;**B** with a nearly identical relationship observed in human microglia (green, IBA1) in AD postmortem brains; **C** Quantification of PLIN2 in GFP TREM2-R47H vs. GFP TREM2 xMGs (green, GFP; red, PLIN2) surrounding amyloid plaques (blue, Amylo-glo) in 7-month old 5X-hCSF1 reveal (**D**) a significant reduction in total PLIN2 area in R47H xMG transplanted mice (**E**) with no significant differences in total plaque area, **F** a significant reduction of the percentage of PLIN2 area normalized to plaque area was also detected in R47H xMG transplanted mice; **G** with no significant difference in the number of PLIN2^+^GFP xMGs in vicinity to plaques; Scale Bars at 10 mm in **A**, 5 mm in **B**, and 20 mm in **C**, *n*=3-4; 5-6 images per mouse; * *P*< 0.05; ** *P* < 0.01

**Fig. 3:**
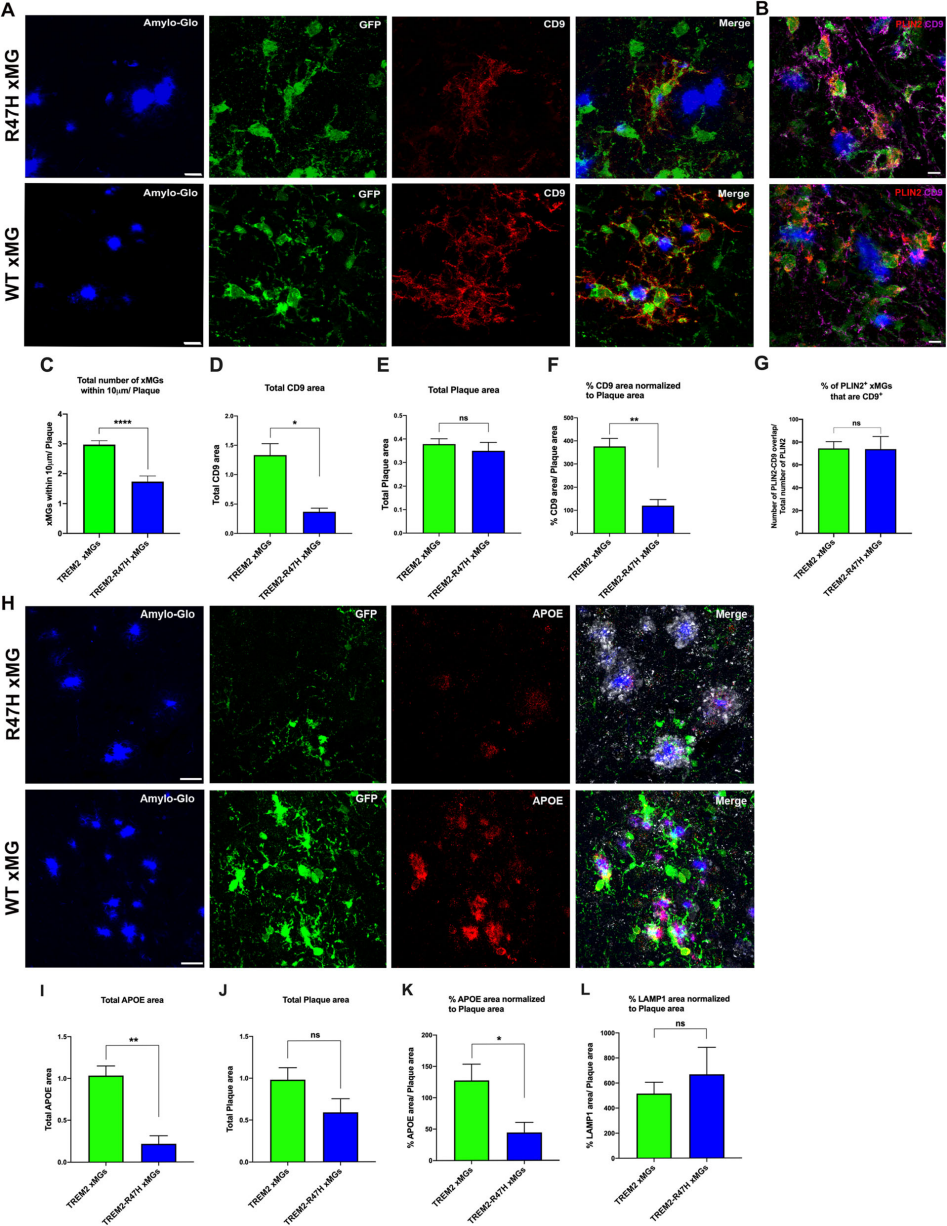
xMGs expressing TREM2 upregulate activation markers around plaques, but less so in TREM2-R47H xMGs. **A** Quantification of xMG proximity to amyloid plaques (blue, Amylo-glo) in 7-month old 5X-hCSF1 revealed a reduction of plaque-associated GFP TREM2-R47H xMGs vs. GFP TREM2 xMGs (green, GFP; red, CD9), **C** within 10 mm per plaque; **D** A significant reduction in total levels of CD9, **E** without a significant difference in total plaque load, and **F** a significant reduction of the percentage of CD9 area normalized to plaque area was observed in TREM2-R47H vs. TREM2 xMGs. **B, G** PLIN2 (red) accumulating GFP xMGs (green) express the DAM-specific marker CD9 (purple) near plaques (blue, Amylo-Glo); **H** GFP TREM2-R47H vs. TREM2 xMGs (green, GFP; Red, APOE; white, LAMP1) reveal a significant reduction in total APOE area (**I**), with no significant difference in total plaque load (**J**), and a significant reduction in APOE area normalized to plaque area (K). An increased trend of the percentage of LAMP1 normalized to plaque area (**L**) was observed in TREM2-R47H xMGs without reaching significance (*P*=0.5635). Scale Bars at 10 mm in A and 20 mm in H; * *P* < 0.05; ** *P* < 0.01; **** *P* < 0.0001; *n*=3-4 mice per genotype; 5-6 images per mouse; Confocal images **B**(scales at 5 mm); *n*=3-4 mice per genotype; 3-4 images per mouse

To further confirm whether DAM xMGs accumulate lipids, we next stained brain cryosections of 7-month old chimeric AD mice transplanted with GFP TREM2 xMGs against the lipid droplet surface protein PLIN2 [34]. Indeed, results show particularly in the plaque-dense subiculum and CA1 regions of the hippocampus, that plaque-associated xMGs accumulate numerous lipid droplets (LDs), which was not observed in microglia distant from plaques (Fig. 2A). Importantly, PLIN2 staining of AD postmortem brains also showed accumulation of LDs within microglia surrounding plaques, further supporting the utility of this chimeric approach to model human disease (Fig. 2B). Interestingly, quantification of LDs (PLIN2) in TREM2-R47H vs. TREM2 xMGs revealed a significant reduction in the total PLIN2 area in TREM2-R47H xMGs and the percentage of PLIN2 area normalized to plaques, without a significant difference in total plaque load (Fig. 2C-F). These novel results indicate that LD accumulation occurs in xMGs in the vicinity of plaques in AD chimeric mice, but is reduced in isogenic TREM2-R47H xMGs. To address whether this reduction might be due to a reduced capacity of R47H xMGs to form lipid droplets near plaques, we next quantified the percentage of microglia in the vicinity of plaques that are positive for PLIN2, which revealed no significant difference between WT and R47H xMGs (Fig. 2G). This data demonstrates that WT and R47H xMGs are equally capable of forming lipid droplets in the vicinity of plaques. Subsequently, as microglial LD accumulation was mainly pronounced in plaque-bound xMGs, we further determined whether iPSC-derived TREM2-R47H xMGs exhibit an overall reduction in their reactivity to amyloid pathology by quantifying the proximity of human microglia to plaques, expression of the DAM-specific marker CD9, and secretion of APOE by human microglia. In agreement with recent findings from transgenic mouse models [21] and human postmortem R47H brain [18], our results show a significant reduction in the number of TREM2-R47H DAM xMGs within 10 μm of fibrillar amyloid plaques (Fig. 3A, C), together with a significant reduction in levels of the DAM marker CD9, and the proportion of CD9 immunoreactivity normalized to total plaque load, with no difference detected in total plaque area (Fig. 3D-F). Furthermore, co-staining of PLIN2 and CD9 revealed PLIN2 expression indeed mainly occurs in CD9 + DAM xMGs (Fig. 3B, G). These data indicate the reduction in total PLIN2 area observed in R47H xMG transplanted mice is due to a reduced number of DAM xMGs, but not a reduced potential of R47H xMGs to accumulate lipid droplets. In addition, a significant reduction in total levels of human APOE and percentage of APOE normalized to total plaque burden were also observed in TREM2-R47H vs. TREM2 xMGs (Fig. 3H-K). Interestingly, human APOE, as detected by a human-specific antibody, was primarily observed decorating amyloid plaques. In this respect, reduced plaque-associated APOE has recently been observed in TREM2 knockout mice [42]. In addition, a trend towards increased LAMP1 immunoreactive dystrophic neurite area normalized to plaque burden was observed in mutant xMGs, but this finding did not reach significance (Fig. 3L). Lastly, R47H xMGs exhibited no significant increase in the homeostatic marker P2RY12 and levels of P2RY12 normalized to plaque burden, but did reveal a trend towards decreased levels of HLA-DR and percentage of HLA-DR area normalized to plaque burden (Additional File 3A-F). Altogether, these results indicate reduced reactivity of TREM2-R47H xMGs to amyloid plaques in chimeric 5X-hCSF1 mice.

In conclusion, our results indicate that human iPSC-derived DAM microglia adopt a transcriptomic signature in AD mice that mimics that of atherosclerotic foam cells and accumulate LDs, which are reduced in TREM2-R47H mutant microglia. Interestingly, *in vitro* the R47H mutation exacerbates microglial lipid accumulation [37]. However, our data indicate that *in vivo*, LD accumulation occurs particularly in the DAM-subset and is thus dependent on microglial reactivity and proximity to plaques, which is impaired by the R47H mutation. Moreover, lipid droplet accumulation was also observed in plaque-associated microglia in postmortem AD brains, further supporting the use of this chimeric approach to model human disease. Whether plaques induce LD accumulation in human microglia via a direct (amyloid uptake) or indirect manner (localized plaque-induced toxicity and cell death) or a combination of the two, remains unknown. However, the observation that LD accumulation occurs secondary to plaque proximity *in vivo* suggests that it will be important to determine whether TREM2 stimulating antibodies or other TREM2 activating approaches that increase microglial association with plaques [43], could in turn affect LD accumulation in xMGs. In this respect, accumulation of LDs in aged microglia impaired their functionality, and *in vitro* pharmacological inhibition of lipid droplet accumulation was able to restore microglial phagocytic capacity [34]. Altogether, the observation that both TREM2 wild-type and mutant human DAM xMGs accumulate LDs around plaques further demonstrates the importance of microglial lipid metabolism in AD and suggests that it will be of great future interest to determine how inhibition of LD accumulation and/or induction of microglial lipid metabolism can influence Alzheimer’s pathology and perhaps uncover promising new therapeutic avenues.

## Methods

### Contact for reagent and resource sharing

Further information and requests for resources and reagents should be directed to and will be fulfilled by Mathew Blurton-Jones (mblurton@uci.edu).

### Animals

All animal procedures were conducted in accordance with the guidelines set forth by the National Institutes of Health and the University of California, Irvine Institutional Animal Care and Use Committee. The M-CSFh mouse line (hCSF1) was purchased form Jackson Laboratories (stock # 017708) and includes deletion of Rag2 and Il2rγ and humanized M-CSFh [44], which are necessary for human microglial engraftment. The 5xFAD-hCSF1 (5X-hCSF1) model was created by backcrossing the M-CSFh mouse with the well-established 5xFAD transgenic model which overexpress co-integrated transgenes for Familial Alzheimer’s Disease (FAD) mutant APP (Swedish, Florida, and London) and mutant FAD PS1 (M146L and L286V) [45].

### Maintenance and acquisition of iPSC lines

Maintenance of all iPSC lines involved culturing in feeder-free conditions in complete TeSR™-E8™medium (Stemcell Technologies), in a humidified incubator (5 % CO2, 37 °C), with medium changed daily. Passaging was performed every 7–8 days using ReLeSR (Stemcell Technologies) and cells were plated onto 6-well plates (Corning), coated with growth factor-reduced Matrigel (1 mg/mL; BD Biosciences), in TeSR™-E8™medium, supplemented with 0.5µM Thiazovivin (Stemcell Technologies) for the first 24 h post-passage. The pluripotency, karyotype, and sterility of all iPSC lines was confirmed via trilineage differentiation (Stem Cell Tech.), array comparative genomic hybridization (aCGH performed by Cell Line Genetics), and MycoAlert (Lonza) testing.

The GFP-expressing iPSC line was purchased from Coriell (AICS-0036 GFP line) and was generated by CRISPR modification of the parental WTC11 line to insert mEGFP into the AAVS1 safe harbor locus (chromosomal location 19q13.4-qter) under the control of a CAGG promoter. More information on this line can be found here: https://www.coriell.org/0/PDF/Allen/ipsc/AICS-0036-006_CofA.pdf. We used CRISPR-Cas9 modification to then introduce the TREM2-R47H homozygous mutation into the AICS-0036 GFP line (Additional File 2): 2 × 10^5^ induced pluripotent stem cells were isolated following Accutase enzymatic digestion for 3 min at 37 ^o^C. Cells were resuspended in 100 µL nucleofection buffer from Human Stem Cell Nucleofector™ Kit 2 (Lonza). The suspension was combined with 2µM ssODN template (IDTDNA) and 50 µg of RNP complex formed by incubating Alt-R® S.p. HiFi Cas9 Nuclease V3 (IDTDNA) with fused crRNA:tracrRNA (IDTDNA) duplex for 15 min at 23 ^o^C. The suspension was transferred to the Amaxa Nucleofector cuvette and transfected using program B-016. Cells were plated in TeSR™-E8™ (STEMCELL Technologies) media with 0.25 µM Thiazovivin (STEMCELL Technologies) and CloneR™ (STEMCELL Technologies) supplement overnight to recover. Cells were digested the following day with Accutase and mechanically single-cell plated to 96-well plates in TeSR™-E8™ media with 0.25 µM Thiazovivin and CloneR™ for clonal isolation and expansion. Genomic DNA was extracted using Extracta DNA prep for PCR (Quantabio) from a sample of each clone upon passage and amplified for sequencing using Taq PCR Master Mix (ThermoFisher Scientific) at the cut site. PCR product from promising clones was transformed using TOPO™ TA Cloning™ Kit for Subcloning, with One Shot™ TOP10 (ThermoFisher Scientific) for allele-specific sequencing. Comparison of GFP TREM2-R47H with its isogenic control allowed the analysis of the effect of the homozygous TREM2-R47H mutation independent of possible genetic modifiers.

### Differentiation of Hematopoietic Progenitor Cells from iPSCs

iPSC-derived Hematopoietic Progenitor Cells (HPCs) were differentiated according to our published protocol [46]. To begin HPC differentiation, iPSCs were passaged in mTeSR-E8 to achieve a density of 40–60 colonies per 6-well. On day 0, cells were transferred to Medium A from the STEMdiff™ Hematopoietic Kit (Stem Cell Technologies). On day 3, flattened endothelial cell colonies were transferred to Medium B and cells remained in medium B for 7 additional days while HPCs began to lift off the colonies. On day 11, non-adherent CD43 + HPCs were collected by removing medium and cells with a pipette. At this point, HPCs can be frozen in Bambanker (Wako) and stored in liquid nitrogen at a concentration of 4 million cells/mL. Cells used for early-postnatal HPC transplantation were thawed and resuspended at 50 K cells/µL in 1X DPBS (low Ca^2+^, low Mg^2+^).

### Early Postnatal Intracerebroventricular Transplantation of HPCs

P1 to P2 5X-hCSF1 mice were placed in a clean cage over a heating pad with a nestlet from the home cage to maintain the mother’s scent. Female pups were then placed on ice for 2–3 min to induce hypothermic anesthesia. Free-hand transplantation was performed using a 30-gauge needle affixed to a 10µL Hamilton syringe, mice received 1µL of HPCs suspended in sterile 1X DPBS at 50 K cells/µL at each injection site (8 sites) totaling 400 K cells/pup. Bilateral injections were performed at 2/5th of the distance from the lambda suture to each eye, injecting into the lateral ventricles at 3mm and into the overlying anterior cortex at 1 mm, and into the posterior cortex in line with the forebrain injection sites, and perpendicular to lambda at a 45° angle. Transplanted pups were then returned to their home cages and weaned at P21. For further details and validation of this chimeric approach please see: [38].

### Tissue dissociation for scRNA-seq

All steps were performed on ice or at 4 °C with ice cold reagents and all centrifuge steps were performed for 10 min at 400xg with full brake and acceleration unless otherwise stated. Anesthetized mice were intracardially perfused with 1X DPBS, half brains were dissected, the cerebellum was removed, and tissue was stored in RPMI 1640 until subsequent perfusions were completed. Brains were manually homogenized using a 7mL Dounce homogenizer by adding 4mL of RPMI 1640 and performing 10 strokes with the “loose” pestle followed by 10 strokes with the “tight” pestle. Samples were then run through a pre-soaked 70 μm filter and the filter was washed with 10 mL of RPMI 1640. The sample was pelleted by centrifugation and myelin was removed by resuspension in 30 % Percoll overlaid with 2 mL of 1X DPBS centrifuged at 400xg for 20 min with acceleration and brake set to 0. The myelin band and supernatant were discarded and cell pellets were resuspended in 80 µL MACS buffer (0.5 % BSA in 1X DPBS) + 20 µL Mouse Cell Removal beads (Miltenyi) and incubated at 4 °C for 15 min. Magnetically labelled mouse cells were separated using LS columns and the MidiMACs separator (Miltenyi) while the unlabeled human cells were collected in the flow through. Human cells were then pelleted by centrifugation and dead cells were magnetically removed using the Dead Cell Removal kit, Annexin V (Stem Cell Technologies) by resuspending the pellets in 100 µL of buffer (2 % BSA + 1mM CaCl_2_ in 1X PBS) in 5 mL polystyrene round-bottom tubes and the following manufacturer protocol. Live cells were centrifuged, resuspended in 50–100 µL of MACS buffer, and concentrations were determined by counting on a hemocytometer. Final cell concentrations were then adjusted to 900-1,000 cells/µL.

### scRNA-seq library preparation and sequencing

scRNA-seq library preparation was performed according to the 10X Genomics Chromium Single Cell 3’ Reagents kit v3 user guide except that sample volumes containing 25,000 cells were loaded onto the 10X Genomics flow cell in order to capture ~ 10,000 total cells. The 10X Genomics workflow was then followed according to the manufacturer protocol and libraries were pooled at equimolar concentrations for sequencing on an Illumina NovaSeq 6000, targeting ~ 50,000 reads per cell. FASTQ files were aligned to both the human GRCh38 transcriptome (Ensembl release 95; [47]) using the CellRanger v3.0.2 count command, with the expected cells set to 10,000 and no secondary analysis performed.

### scRNA-seq Data Visualization and Differential Gene Analysis

UMI count tables were read into Seurat (v3) [48] for preprocessing and clustering analysis (Additional File 1). Initial QC was performed by log normalizing and scaling (default settings) each dataset followed by PCA performed using all genes in the dataset. Seurat’s ‘ElbowPlot’ function was used to select principal components (PCs) to be used for clustering along with a resolution parameter of 0.35 and clusters identified as being doublets, gene poor, or dividing were removed from the dataset prior to downstream analysis. Secondary QC cutoffs were then applied to retain only cells with less than 27.5 % ribosomal genes, 12.5 % mitochondrial genes, greater than 500 genes but less than double the median gene count, and greater than 500 UMI but less than double the median UMI count. Additionally, subsequent analysis identified a small cell population (179 cells; Additional File 1) primarily present in only a single sample, which were removed as the cluster did not appear to be biologically relevant.

Cells passing QC for each sample were then merged using Seurat’s ‘merge’ function and datasets were processed using Seurat’s integrated analysis workflow [49]. In short, samples from individual mice were integrated using the ‘FindIntegationAnchors’ and ‘IntegrateData’ commands using dimensions 1:25. Datasets were then scaled and sources of technical variation were regressed out (library size differences, percent ribosomal genes, and percent mitochondrial genes) and PCA was performed using Seurat’s ‘RunPCA’ command. A shared nearest neighbor (SNN) plot was generated using Seurat’s ‘FindNeighbors’ function using PCs 1:15 as input, clustering was performed using the ‘FindClusters’ function and a resolution parameter of 0.3, and dimension reduction was performed using the ‘RunUMAP’ function with the same PCs used for generating the SNN plot. Differentially expressed genes were determined between clusters using the ‘FindAllMarkers’ function, which employs a Wilcoxon Rank Sum Test, with and FDR cutoff of 0.01, an LFC cutoff of 0.25, and the requirement that the gene be expressed in at least 10 % of the cluster and clusters were labeled according to manual curation of the differential gene lists. Differentially expressed genes between genotypes across all cells were identified using the “FindMarkers” function in Seurat 3.2.1, using a log-FC cutoff of 0.001 and adjusted *p*-value < 0.01 (Fig. 1E, H, I). The y-axis of the violin plots depicted in Fig. 1I feature counts per cell divided by the total counts for the cell and multiplied by a scale factor of 10,000, then natural-log transformed using log1p, using the “NormalizeData” function in Seurat.

### Immunohistochemistry and Confocal Microscopy

Animals were administered Euthasol and monitored for loss of consciousness. Once animals no longer responded to toe pinch, mice were intracardially perfused with ice cold 1X DPBS. If xMGs were being isolated from half brains, the remaining half brain was drop fixed in 4 % (w/v) PFA for 48 hours, otherwise, the mice were intracardially perfused with 4 % PFA and post-fixed for 24 hours. Samples were then cryoprotected in 30 % (w/v) sucrose until the tissue sank in the solution. Brains were then cut coronally at a section thickness of 40um on a sliding microtome cooled to -79°C. Tissue sections were collected as free-floating sections in PBS with 0.05 % sodium azide. For staining, tissue was blocked for 1 hour in 1X PBS, 0.2 % Triton X-100, and 10 % goat serum. Immediately following blocking, sections were stained for amyloid plaques using the UV ThioflavinS analog Amylo-Glo (Biosensis, TR-300-AG, 1:100) for 20’ at RT on a shaker in the dark. Next, sections were placed in primary antibodies diluted in 1X PBS with 1 % goat serum and incubated overnight on a shaker at 4 °C (GFP-ch, Millipore AB16901, 1:500; CD9-ms clone HI9a, Biolegend 312,102, 1:200; APOE-Rb, Thermofisher PA5-27088, 1:1000; LAMP1-Rt, Abcam ab25245, 1:200; PLIN2-Gp, Fitzgerald 20R-AP002; 1:500, HLA-DR-ms (Invitrogen 14-9956-82; 1:200), P2RY12-Rb (Novus Biologicals NBP233870; 1:400, 1 h RT). Sections were then incubated in conjugated secondary antibodies (AlexaFluor Antibodies, Life Technologies, 1:400) for 1 h, before washing and mounting on microscope slides. Immunofluorescent sections were then visualized and captured using an Olympus FV3000 confocal microscope. In some cases, brightness and contrast settings of confocal images were slightly adjusted to reveal fine structures and morphology. Importantly, no adjustments were made to any images used for quantification. Human AD brain tissue from the hippocampus used in this project were provided by the University of California Alzheimer’s Disease Research Center (UCI-ADRC) and the Institute for Memory Impairments and Neurological Disorders. AD brain sections were stained using Iba1-Rb (Wako, 1:200), PLIN2-Gp (Fitzgerald 20R-AP002, 1:200) and Amylo-Glo (Biosensis, TR-300-AG, 1:100).

### IHC Quantification

HPCs derived from isogenic wild-type and TREM2-R47H GFP iPSCs (Coriell, AICS-0036) were transplanted into 5X-hCSF1 female pups. 7-months later, immunohistochemistry was performed, and confocal Z-stacks collected within the retrosplenial granular cortex (proximity to plaques, plaque area, CD9 area, APOE area, LAMP1 area, HLA-DR area and P2RY12 area) and within the dorsal aspect of the subiculum and CA1 (PLIN2 area and CD9 area) at 40x magnification using identical confocal settings between TREM2 genotypes (*n* = 4 mice (TREM2 xMGs) *n* = 3 mice (TREM2-R47H xMGs) with 3–6 images per mouse). To study proximity to plaques human microglia locations were detected and quantified through GFP immunofluorescence within 10 μm per plaque (Amylo-Glo, blue) using the Cellsense software on the Olympus FV3000. Ordinary one-way Anova was performed to confirm similar distribution of samples within a given genotype (Additional File 4A-B) in order to combine all values per genotype in a singular distribution and perform Welch’s t test (Fig. 3C). Plaques near the borders of the image were excluded, and for plaques within 10 μm of each other, only the plaque with the highest number of xMGs was included for both genotypes. Plaque area of the plaques included for xMG counts show no significant difference between genotypes (Additional File 4C) and also after normalization of the number of xMGs within 10 μm per plaque to plaque area, the significant reduction in the number of R47H-mutant xMGs remains (Additional File 4D). IMARIS-based quantification of CD9, APOE, LAMP1, PLIN2, HLA-DR, P2RY12 and total plaque load was performed using the “Surfaces” function and were measured by the sum of surfaces for each image. To determine the number of PLIN2^+^ GFP xMGs, the “classification” function of Imaris was used to measure the number of GFP surfaces that overlap with PLIN2 in the vicinity of plaques (Fig. 2G). The number of surfaces that show overlap of CD9 and PLIN2 was determined by using the “classification” function (Fig. 3G).

Unless stated otherwise, data were tested for statistical significance (*P* < 0.05) through Welch’s t-test using Prism 8.

### Statistical analysis

All statistical analyses were performed using either R programming or Prism 8.

## Supplementary information

**Additional file 1. **Sc-RNAseq information table. Data pertaining to the Seurat analysis of the scRNA-seq data contained in Fig. 1. Tables include samples basics and Seurat analysis parameters, cluster barcodes, results of differential gene expression analysis for each cluster, pseudobulk analysis and the foam cell gene signature.

**Additional file 2. ** CRISPR/Cas9 editing to create a homozygous TREM2-R47H mutant human iPSC line. The chromatogram demonstrates CRISPR/Cas9 induced targeting of CGC (Arginine (R)) in the wild-type allele of TREM2 exon 2, to CAC (Histidine (H)) (together with the insertion of some additional silent mutations labeled in red) in both alleles, resulting in a TREM2 homozygous R47H mutation in the commercially available GFP iPSC line from Coriell.

**Additional file 3. ** TREM2-R47H mutant xMGs reveal no significant increase in the levels of P2RY12, but a decreased trend in the expression of HLA-DR compared to TREM2-WT xMGs in 5X-hCSF1. (A) Quantification of HLA-DR and P2RY12 in GFP TREM2-R47H vs. GFP TREM2 xMGs (green, GFP; red, HLA-DR; purple, P2RY12) surrounding amyloid plaques (blue, Amylo-glo) in 7-month old 5X-hCSF1 reveal (B) a trend towards decrease in total HLA-DR area (*P*=0.0789) (C) with no significant differences in total plaque area, (D) a trend towards decrease in the percentage of HLA-DR area normalized to plaque area (*P*=0.1716), and (E) no significant increase in total P2RY12 area (*P*=0.2848) and (F) percentage of P2RY12 area normalized to plaque area (*P*=0.3202) in TREM2-R47H vs. TREM2 xMGs. Scale Bar at 10 mm; *n*=3-4 mice per genotype; 3 images per mouse.

**Additional file 4.** IHC analysis of xMG proximity per Plaque in 5X-hCSF1. Human microglia locations were detected and quantified through GFP immunofluorescence within 10μm per plaque (Amylo-Glo, blue) using the Cellsense software on the Olympus FV3000. (A-B) Ordinary one-way Anova was performed to confirm similar distribution of samples within a given genotype; (C) Plaque area of the plaques included for xMG counts show no significant difference between genotypes; (D) After normalization of the number of xMGs within 10 μm per plaque to plaque area, the significant reduction in the number of R47H-mutant xMGs remains; Unless stated otherwise, data were tested for statistical significance (*P*<0.05) through Welch’s t-test using Prism 8 (**** *P* < 0.0001; *n*= 3-4 mice per genotype; 5-6 images per mouse).

## Data Availability

All data generated during this study are included in this published article.

## Abbreviations

AD: Alzheimer’s disease
DAM: Disease-associated microglia
HPCs: iPSC-derived hematopoietic progenitors
iPSC: Induced pluripotent stem cells
LD: Lipid droplets
LDAM: Lipid droplet-accumulating microglia
scRNA-seq: Single-cell RNA sequencing
TREM2: Triggering receptor expressed on myeloid cells 2
xMG: Xenografted human iPSC-derived microglia
WT: Wild-type
