# Supplementary figures and images for: Plaque-associated human microglia accumulate lipid droplets in a chimeric model of Alzheimer’s disease
Source: Mol Neurodegener. 2021 Jul 23;16:50. doi: 10.1186/s13024-021-00473-0 (PMC8305935; doi:10.1186/s13024-021-00473-0)

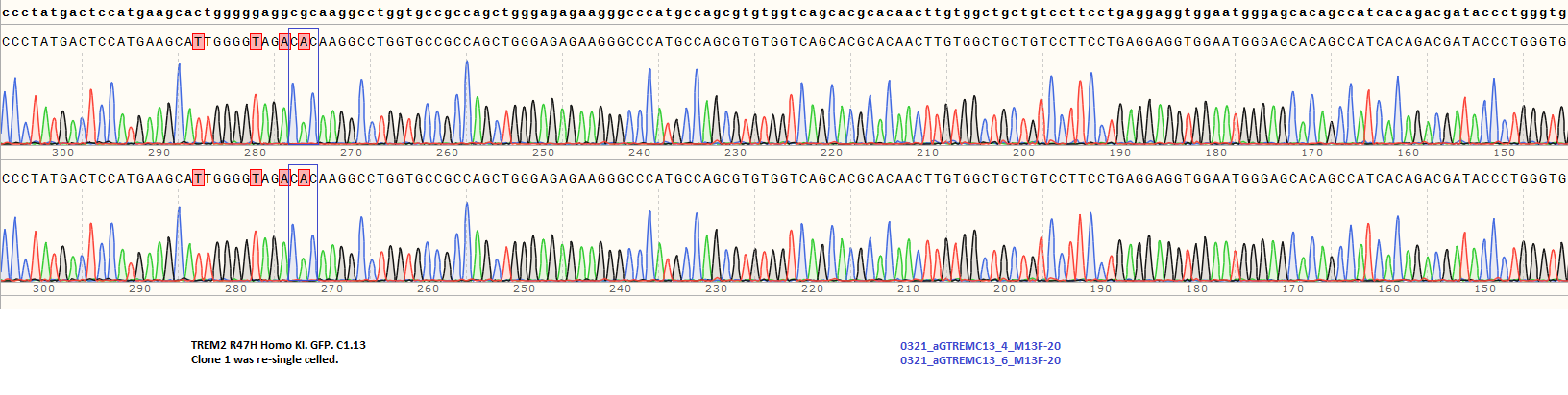

Supplement: Supplementary file 2 — Additional file 2. CRISPR/Cas9 editing to create a homozygous TREM2-R47H mutant human iPSC line. The chromatogram demonstrates CRISPR/Cas9 induced targeting of CGC (Arginine (R)) in the wild-type allele of TREM2 exon 2, to CAC (Histidine (H)) (together with the insertion of some additional silent mutations labeled in red) in both alleles, resulting in a TREM2 homozygous R47H mutation in the commercially available GFP iPSC line from Coriell. [file 13024_2021_473_MOESM2_ESM.png]

**A**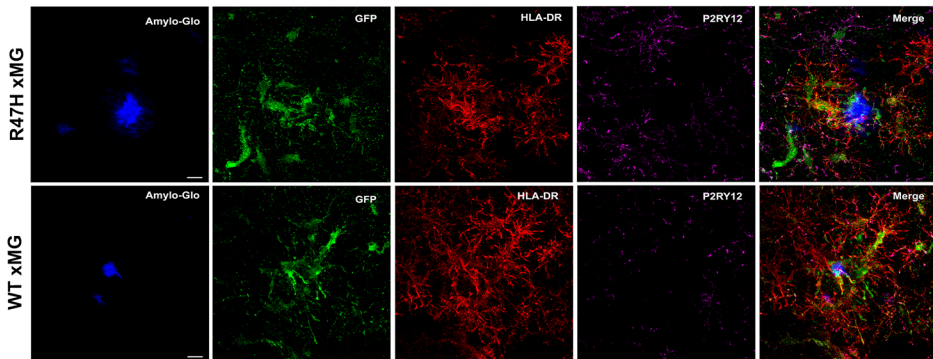**B**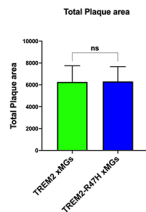**C**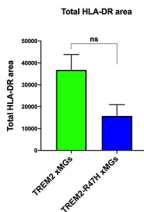**D**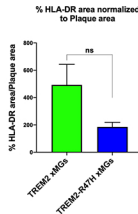**E**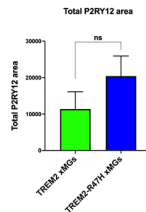**F**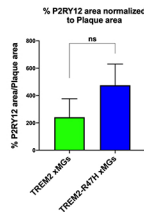

Supplement: Supplementary file 3 — Additional file 3. TREM2-R47H mutant xMGs reveal no significant increase in the levels of P2RY12, but a decreased trend in the expression of HLA-DR compared to TREM2-WT xMGs in 5X-hCSF1. (A) Quantification of HLA-DR and P2RY12 in GFP TREM2-R47H vs. GFP TREM2 xMGs (green, GFP; red, HLA-DR; purple, P2RY12) surrounding amyloid plaques (blue, Amylo-glo) in 7-month old 5X-hCSF1 reveal (B) a trend towards decrease in total HLA-DR area (P=0.0789) (C) with no significant differences in total plaque area, (D) a trend towards decrease in the percentage of HLA-DR area normalized to plaque area (P=0.1716), and (E) no significant increase in total P2RY12 area (P=0.2848) and (F) percentage of P2RY12 area normalized to plaque area (P=0.3202) in TREM2-R47H vs. TREM2 xMGs. Scale Bar at 10 mm; n=3-4 mice per genotype; 3 images per mouse. [file 13024_2021_473_MOESM3_ESM.pdf]

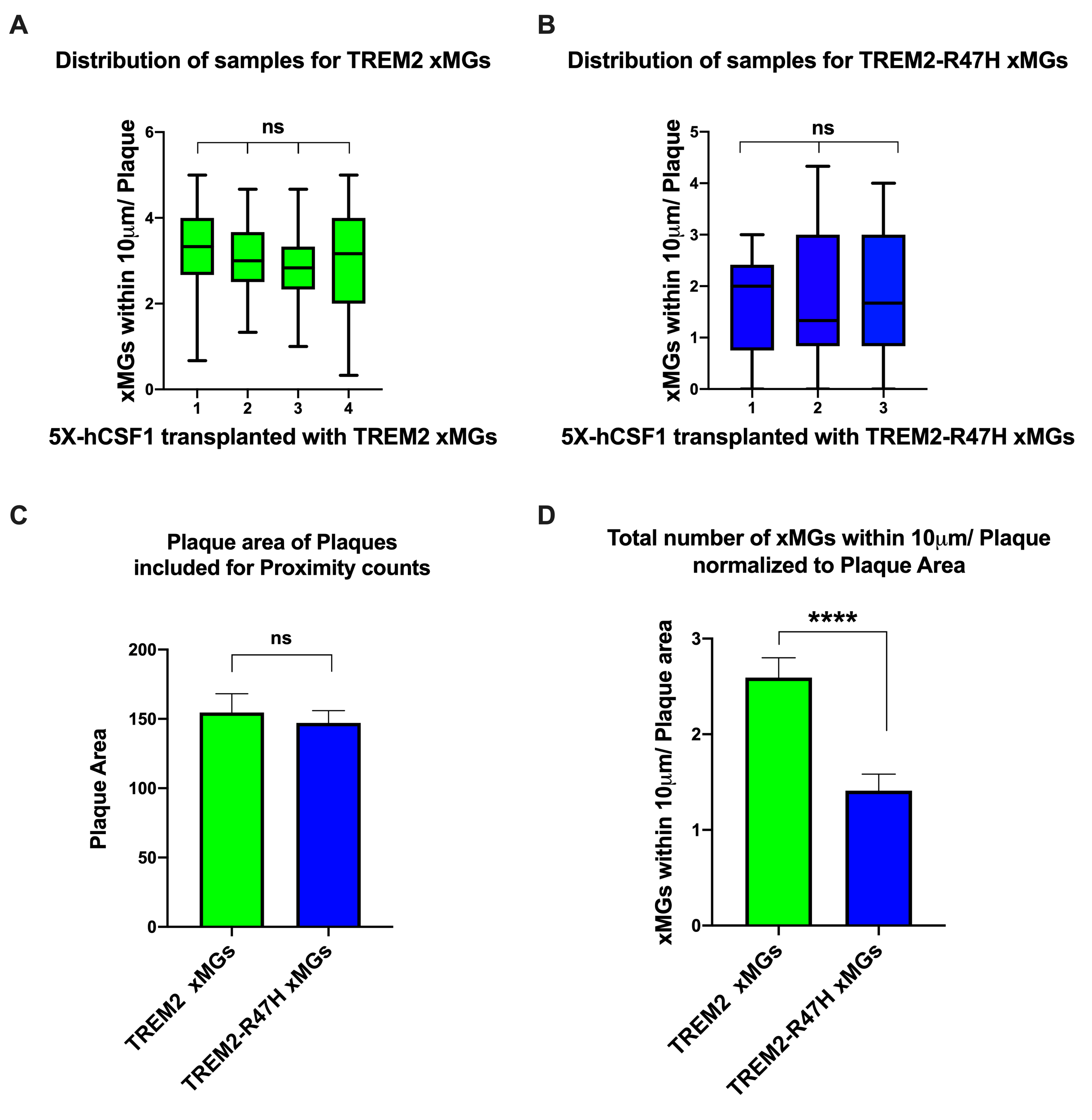

Supplement: Supplementary file 4 — Additional file 4. IHC analysis of xMG proximity per Plaque in 5X-hCSF1. Human microglia locations were detected and quantified through GFP immunofluorescence within 10μm per plaque (Amylo-Glo, blue) using the Cellsense software on the Olympus FV3000. (A-B) Ordinary one-way Anova was performed to confirm similar distribution of samples within a given genotype; (C) Plaque area of the plaques included for xMG counts show no significant difference between genotypes; (D) After normalization of the number of xMGs within 10 μm per plaque to plaque area, the significant reduction in the number of R47H-mutant xMGs remains; Unless stated otherwise, data were tested for statistical significance (P<0.05) through Welch’s t-test using Prism 8 (**** P < 0.0001; n= 3-4 mice per genotype; 5-6 images per mouse). [file 13024_2021_473_MOESM4_ESM.png]
